# Correction: Controlling the preferential motion of chiral molecular walkers on a surface

**DOI:** 10.1039/c9sc90124h

**Published:** 2019-06-05

**Authors:** David Abbasi-Pérez, Hongqian Sang, Lluïsa Pérez-García, Andrea Floris, David B. Amabilino, Rasmita Raval, J. Manuel Recio, Lev Kantorovich

**Affiliations:** a Department of Physics , King’s College London , London , WC2R 2LS , UK . Email: stqa8038@kcl.ac.uk; b Institute for Interdisciplinary Research , Jianghan University , Wuhan 430056 , China; c School of Pharmacy , University of Nottingham , University Park , Nottingham , NG7 2RD , UK; d School of Chemistry , University of Lincoln , Brayford Pool , Lincoln LN6 7TS , UK; e School of Chemistry , GSK Carbon Neutral Lab. for Sustainable Chemistry , University of Nottingham , Triumph Road , NG7 2TU , UK; f Surface Science Research Centre , Department of Chemistry , University of Liverpool , Liverpool L69 3BX , UK; g MALTA-Consolider Team and Department of Analytical and Physical Chemistry , Universidad de Oviedo , Oviedo , 33006 , Spain

## Abstract

Correction for ‘Controlling the preferential motion of chiral molecular walkers on a surface’ by David Abbasi-Pérez *et al.*, *Chem. Sci.*, 2019, DOI: ; 10.1039/c9sc01135h.



## 

We regret that in the original article, one of the funding grants was not acknowledged. The correct sentence should read as follows:

This research was supported by the UK EPSRC grant EP/J019844/1 and in part by EP/N023587/1 and EP/M005178/1.

In addition, on column 2 of page 7, the phrase “…the (*S*) molecule moves to the right (*E*_eff_ > 0) slower than (*R*) in the case of the opposite direction of the field…” should be corrected to “…the (*S*) molecule moves to the right (*E*_eff_ > 0) slower than (*R*); in the case of the opposite direction of the field…”.

The Royal Society of Chemistry apologises for these errors and any consequent inconvenience to authors and readers.

